# Effectiveness of Social Needs Screening and Interventions in Clinical Settings on Utilization, Cost, and Clinical Outcomes: A Systematic Review

**DOI:** 10.1089/heq.2022.0010

**Published:** 2022-06-24

**Authors:** Alice F. Yan, Zhuo Chen, Yang Wang, Jennifer A. Campbell, Qian-Li Xue, Michelle Y. Williams, Lance S. Weinhardt, Leonard E. Egede

**Affiliations:** ^1^ Center for Advancing Population Science (CAPS), Division of Internal Medicine, Department of Medicine, Medical College of Wisconsin, Wauwatosa, Wisconsin, USA.; ^2^ Department of Health Policy and Management, College of Public Health, University of Georgia, Athens, Georgia, USA.; ^3^ China Center for Health Development Studies, Peking University, Beijing, China.; ^4^ Division of Geriatric Medicine and Gerontology, Department of Medicine and the Center on Aging and Health, School of Medicine, The Johns Hopkins University, Baltimore, Maryland, USA.; ^5^ Division of Research, Nursing and Patient Care Services, Stanford Health Care, Palo Alto, California, USA.; ^6^ Joseph J. Zilber School of Public Health, University of Wisconsin Milwaukee, Milwaukee, Wisconsin, USA.

**Keywords:** social needs, screening, intervention, health care utilization and cost, clinical outcomes, systematic review

## Abstract

**Objective:**

This systematic review examined and synthesized peer-reviewed research studies that reported the process of integrating social determinants of health (SDOH) or social needs screening into electronic health records (EHRs) and the intervention effects in the United States.

**Methods:**

Following PRISMA (Preferred Reporting Items for Systematic Reviews and Meta-Analysis) guidelines, a systematic search of Scopus, Web of Science Core Collection, MEDLINE, and Cochrane Central Register of Clinical Trials was performed. English language peer-reviewed studies that reported the process of integrating SDOH or social needs screening into EHRs within the U.S. health systems and published between January 2015 and December 2021 were included. The review focused on process measures, social needs changes, health outcomes, and health care cost and utilization.

**Results:**

In total, 28 studies were included, and half were randomized controlled trials. The majority of the studies targeted multiple SDOH domains. The interventions vary by the levels of intensity of their approaches and heterogeneities in outcome measures. Most studies (82%, *n*=23) reported the findings related to the process measures, and nearly half (43%, *n*=12) reported outcomes related to social needs. By contrast, only 39% (*n*=11) and 32% (*n*=9) of the studies reported health outcomes and impact on health care cost and utilization, respectively. Findings on patients' social needs change demonstrated improved access to resources. However, findings were mixed on intervention effects on health and health care cost and utilization. We also identified gaps in implementation challenges to be overcome.

**Conclusion:**

Our review supports the current policy efforts to increase U.S. health systems' investment toward directly addressing SDOH. While effective interventions can be more complex or resource intensive than an online referral, health care organizations hoping to achieve health equity and improve population health must commit the effort and investment required to achieve this goal.

## Introduction

In Sir Michael Marmot's landmark Whitehall study (1978),^1^ he offered primary evidence of the dose–response association between the health outcomes and socioeconomic status of those in the British Civil Service. As a result of this, our collective understanding of the impact of social, economic, behavioral, and environmental factors on individuals' health has grown. Social determinants of health (SDOH) are defined by The World Health Organization Commission^2–4^ as the “conditions in which people are born, grow, live, work, and age.” This landmark document outlined three recommendations for improving the conditions experienced by individuals: (1) address unbalanced distributions of power, money, and resources; (2) measure the problem; and (3) evaluate the impact of actions.

United States Health systems' interest in addressing SDOH has increased markedly in recent years, as exemplified by new attention from policymakers and researchers. For example, in the 2010 Patient Protection and Affordable Care Act, a mandate was implemented that requires nonprofit hospitals to participate in community-level planning to improve community health and to conduct Community Health Needs Assessments every 3 years.^5^ Mounting evidence shows that SDOH account for substantially more variation in health outcomes than medical care.^3,6^ A social need is described as the need of an individual as a result of SDOH.^7^ Thus, investment in addressing social need(s) through social services (e.g., housing, financial resources, food access), care coordination, and community outreach can positively impact health outcomes and reduce health care spending.^8^

As the U.S. health care systems move toward value-based models that incentivize positive results, they have started the momentum to seek out new ways to collect data on SDOHs from patients' electronic health records (EHRs) and incorporate SDOH-related screen and referral, specifically to screen for social needs, and provide a tailored intervention into routine care among patients who are identified to have social needs.^9,10^

Prior reviews have explored the impact of screening for SDOH in clinical care settings,^11^ using SDOH data found in EHR to predict risk,^12^ reliability, and validity of screening tools,^13^ and effectiveness of intervention in addressing multiple domains of SDOH or a particular social need in the health care setting,^14,15^ as well as evidence, implementation, challenges, and opportunities.^16^ However, at least four questions related to the implementation and effectiveness of interventions to address domains of SDOH remain not fully answered. First, is screening for social needs in clinical settings effective? Few studies provided clear descriptions of the clinical workflow involved in screening for and integrating SDOH in EHRs, and the subsequent interventions either through social prescriptions, such as health professionals working with social workers or community connectors to refer patients who have social needs with community resources or social services. Social prescription can be administered through referrals or direct support within health care settings to address patients' social needs related to SDOH.

Second, are social needs interventions delivered in clinical settings effective at reducing cost/utilization and improving clinical outcomes? To our knowledge, and documented in other reviews,^14,16,17^ limited studies reported intervention effects on a full range of outcomes, including (1) process measures; (2) short-term outcome directly related to social needs (e.g., level of need and what percentage of these needs were met), (3) intermediate outcome or impact on health outcomes, and (4) long-term outcome or impact on health care cost or utilization. Within these studies, less than half reported impacts of such intervention on SDOH (48%), health impact (30%), and health care cost or utilization impacts (27%), respectively.^14^ Third, what are the components of effective social needs interventions in clinical settings? Fourth, what are barriers, facilitators, and resources needed to implement effective social needs interventions in clinical settings?

To address these gaps, this systematic review examined and synthesized peer-reviewed research studies that reported the process of integrating SDOH or social needs screening into EHRs for documenting social needs and the intervention effects on a full range of outcomes, including process measures, impacts on social needs, health outcomes, and health care cost and utilization. In addition, this review discussed current gaps and opportunities to implement effective social needs interventions in future research.

## Methods

### Data sources and search strategies

Following the PRISMA (Preferred Reporting Items for Systematic Reviews and Meta-Analysis) checklist and guidelines, a systematic review was conducted to summarize the existing studies.^18^ Four major medical and public health databases were used in this study, including Scopus, Web of Science Core Collection, MEDLINE (Ovid), and Cochrane Central Register of Clinical Trials (Wiley). Limits set included articles in English, conducted in the United States, and published between January 1, 2015 (the year immediately after collecting SDOH in health care systems was recommended by the Institute of Medicine) and December 2021.

**FIG. 1.**
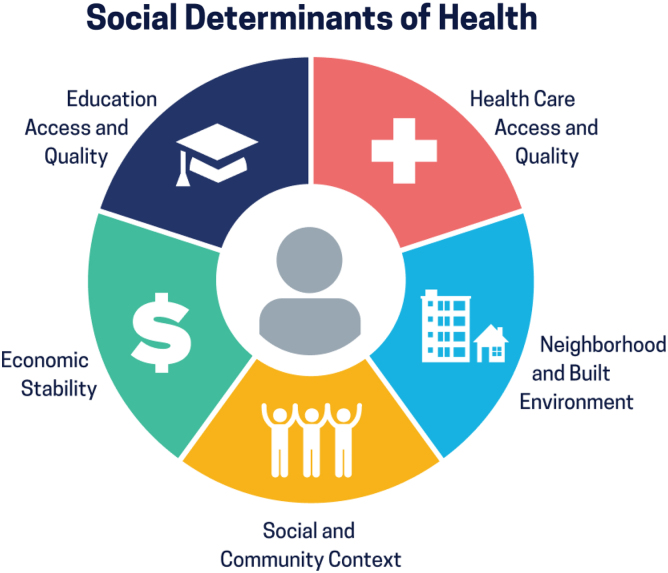
Healthy People 2030 social determinants of health framework. SDOH, social determinants of health. Citation of the SDOH graphic: Healthy People 2030, U.S. Department of Health and Human Services, Office of Disease Prevention and Health Promotion. Retrieved December 12, 2021, from https://health.gov/healthypeople/objectives-and-data/social-determinants-health

The strategy deployed for this search used keywords and medical subject headings (MeSH) combined with database-specific techniques for advanced search. A number of keywords and MeSH terms were identified to represent the topics of SDOH, social needs, screening, and EHRs in health care setting. According to Healthy People 2030^19^ framework, SDOH are categorized into five different domains: “economic stability, education access and quality, health care access and quality, neighborhood and built environment, and social and community context.” Given the current literature and the diverse topics of addressing social needs or SDOH, the Healthy People 2030^19^ framework was used to select articles for inclusion and synthesizing (Fig. 1).

Following the guidelines established above, the keywords and MeSH terms were established for each of the four databases. The strategy for this search is fully detailed in Supplementary Data S1. In sum, 2241 results generated from the literature searches were downloaded into the reference management software Endnote. After removing duplicate articles, 1325 unique publications were uploaded into Rayyan online review software^20^ (https://rayyan.qcri.org/) for subsequent screening. For comprehensiveness, we included additional eight journal articles identified from other sources, such as Google Scholar, and further from a citation search by scanning the references of these articles.

### Study selection: inclusion and exclusion criteria

Two independent reviewers (A.Y. and Y.W.) screened all the results to determine eligibility for this review. Selection was based on whether the study met all six inclusion criteria detailed below. The rationale for inclusion and exclusion criteria aligned with the study objectives.

(1) Published in peer-reviewed academic journals, between January 1, 2015, and December 2021, and in English;
(2) Studies that involved interventions/quality improvement initiatives targeting social risks or domains of SDOH;
(3) Conducted in a U.S. health care setting;
(4) Studies that explicitly mentioned or implied modules/tools embedded into EHR system and described the clinical SDOH-EHR integration as part of the clinical workflow, which also reported some or all of the components of the workflow (i.e., time frame and care team member[s] for administering an assessment/screening, and data entry; identify EHR-documented SDH needs and SDOH referral or intervention; tracking referrals and follow-up);
(5) Studies that examined the impact of such interventions/quality improvement initiatives on a host of outcomes, including process and outcome changes on social needs, health impact, and health care cost or utilization.

Studies were excluded if they were (1) systematic reviews/meta-analyses; (2) opinions, commentaries, or perspectives; (3) qualitative studies; (4) cross-sectional or observational studies that simply used the hospital/clinic EHR system as a tool for extracting data records to investigate the associations between SDOH and health outcomes/health care utilization; or (5) study protocol. Lastly, we excluded studies primarily focused on health care access or outreach programs. Although health care access contributes to health disparities, the focus of this review was health system/clinical programs that integrate SDOH screening (to identify social and economic needs) into an EHR system to address patients' unmet social or financial needs.

### Data extraction and assessment of bias

The data screening and extraction involved a two-phase procedure. The first screening phase was a title/abstract review, and the second phase was a full-text review. Both phases were blinded (one reviewer cannot see the decision of the other) to avoid reviewer bias and conducted in Rayyan. Conflicts on review results were resolved through group consensus. The first phase of screening identified 145 articles for the full-text screening. Following the inclusion and exclusion criteria being applied to the second phase screening, 28 articles were left for synthesis.

**FIG. 2.**
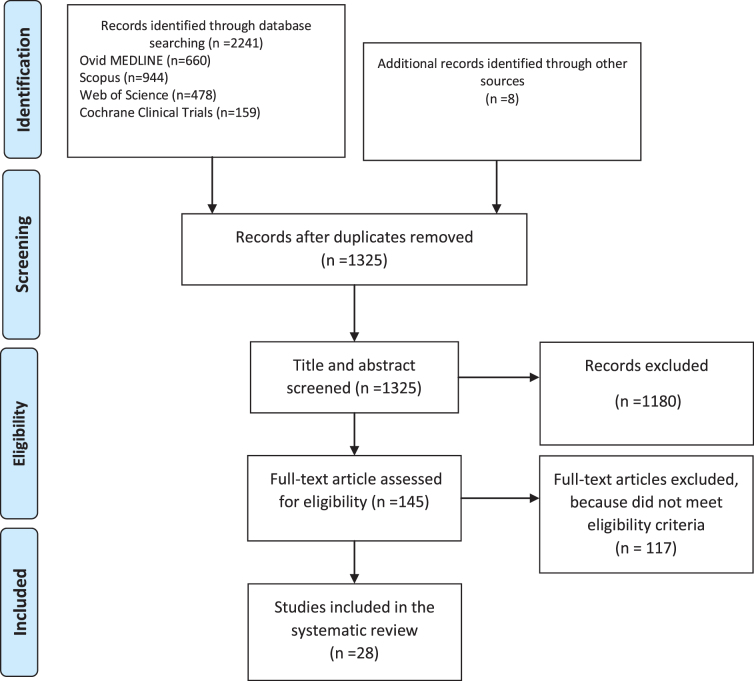
PRISMA flow diagram. PRISMA, Preferred Reporting Items for Systematic Reviews and Meta-Analysis.

Two independent reviewers (A.Y. and Y.W.) extracted the data listed below from each article by following a uniform format: (1) study design, sample characteristics and sample size, study quality; (2) clinical setting; (3) target SDOH (what SDOH were screened and the screening tool[s]); (4) integration into clinical workflow (i.e., screening and data collection); (5) intervention; and (6) outcome measures. The quality of these studies was then graded based on the quality rating of the Grading Recommendations Assessment Development and Evaluation (GRADE).^21^ Then the article results selection in each phase of search and screening processes are provided in Figure 2. PRISMA checklist was presented in Table 1.

**Table 1.** Preferred Reporting Items for Systematic Reviews and Meta-Analysis Checklist

Section/topic	No.	Checklist item	Reported on page number
Title			
Title	1	Identify the report as a systematic review, meta-analysis, or both.	1
Abstract			
Structured summary	2	Provide a structured summary, including, as applicable: background; objectives; data sources; study eligibility criteria, participants, and interventions; study appraisal and synthesis methods; results; limitations; conclusions and implications of key findings; systematic review registration number.	1
Introduction			
Rationale	3	Describe the rationale for the review in the context of what is already known.	1–2
Objectives	4	Provide an explicit statement of questions being addressed with reference to PICOS.	3
Methods			
Protocol and registration	5	Indicate if a review protocol exists, if and where it can be accessed (e.g., Web address), and, if available, provide registration information, including registration number.	n/a
Eligibility criteria	6	Specify study characteristics (e.g., PICOS, length of follow-up) and report characteristics (e.g., years considered, language, publication status) used as criteria for eligibility, giving rationale.	3–5
Information sources	7	Describe all information sources (e.g., databases with dates of coverage, contact with study authors to identify additional studies) in the search and date last searched.	3
Search	8	Present full electronic search strategy for at least one database, including any limits used, such that it could be repeated.	4–5, Suppl 1
Study selection	9	State the process for selecting studies (i.e., screening, eligibility, included in systematic review, and, if applicable, included in the meta-analysis).	4–5, Figure 2
Data collection process	10	Describe method of data extraction from reports (e.g., piloted forms, independently, in duplicate) and any processes for obtaining and confirming data from investigators.	4–5
Data items	11	List and define all variables for which data were sought (e.g., PICOS, funding sources) and any assumptions and simplifications made.	4–5
Risk of bias in individual studies	12	Describe methods used for assessing risk of bias of individual studies (including specification of whether this was done at the study or outcome level), and how this information is to be used in any data synthesis.	5, Table 2
Summary measures	13	State the principal summary measures (e.g., risk ratio, difference in means).	n/a
Synthesis of results	14	Describe the methods of handling data and combining results of studies, if done, including measures of consistency (e.g., *I*^2^) for each meta-analysis.	n/a
Risk of bias across studies	15	Specify any assessment of risk of bias that may affect the cumulative evidence (e.g., publication bias, selective reporting within studies).	n/a
Additional analyses	16	Describe methods of additional analyses (e.g., sensitivity or subgroup analyses, meta-regression), if done, indicating which were prespecified.	n/a
Results			
Study selection	17	Give numbers of studies screened, assessed for eligibility, and included in the review, with reasons for exclusions at each stage, ideally with a flow diagram.	5, Figure 2
Study characteristics	18	For each study, present characteristics for which data were extracted (e.g., study size, PICOS, follow-up period) and provide the citations.	5–6, Table 2
Risk of bias within studies	19	Present data on risk of bias of each study and, if available, any outcome-level assessment (item 12).	Table 2
Results of individual studies	20	For all outcomes considered (benefits or harms), present, for each study: (a) simple summary data for each intervention group (b) effect estimates and confidence intervals, ideally with a forest plot.	6–8, Table 2
Synthesis of results	21	Present results of each meta-analysis done, including confidence intervals and measures of consistency.	n/a
Risk of bias across studies	22	Present results of any assessment of risk of bias across studies (item 15).	n/a
Additional analysis	23	Give results of additional analyses, if done (e.g., sensitivity or subgroup analyses, meta-regression [item 16]).	n/a
Discussion			
Summary of evidence	24	Summarize the main findings, including the strength of evidence for each main outcome; consider their relevance to key groups (e.g., health care providers, users, and policy makers).	9–11
Limitations	25	Discuss limitations at study and outcome level (e.g., risk of bias), and at review level (e.g., incomplete retrieval of identified research, reporting bias).	12
Conclusions	26	Provide a general interpretation of the results in the context of other evidence, and implications for future research.	12
Funding			
Funding	27	Describe sources of funding for the systematic review and other support (e.g., supply of data), role of funders for the systematic review.	n/a

PICOS, participants, interventions, comparisons, outcomes, and study design.

## Results

Twenty-eight studies^22–49^ were included in this systematic review. Their characteristics are summarized in Table 2.

**Table 2.** Key Characteristics and Results of Twenty-Eight Studies Included in the Systematic Review

Author (Year)	Study design, sample characteristics and size, quality	Clinical setting	Target SDOH: what SDOH were screened and the screening tool(s)	Integration into the clinical workflow (i.e., screening and data collection)	Interventions	Outcome measures
Garg et al. (2015)^22^	Study design: RCT (Cluster) Sample: 336 mothers of healthy infants (168 intervention arm and 168 control arm). Quality: high	8 urban CHCs	Use a survey, measured six basic needs (childcare, food security, 18-item U.S. Food Security Scale), household heat, housing instability, parent education, and employment).	The research team provided the self-reported clinical screening instrument to all mothers and attempted to readminister it at subsequent well-child visits	WE CARE, clinic-based screening and referral system (Well-Child Care, Evaluation, Community Resources, Advocacy, Referral, Education)	Process: + Change in number of social referrals, more enrolled in new community resources. SDOH: Greater odds of being employed, children had greater odds of being in childcare; families had greater odds of receiving fuel assistance and lower odds of being in a homeless shelter. Health: None reported Cost/utilization: None reported
Hassan et al. (2015)^23^	Study design: Prospective single group analysis. Sample: 401 youth, 15–25 years of age, from an urban adolescent/young adult clinic Quality: low	Urban hospital-based adolescent and young adult clinic	Youth Risk Behavior Survey, the Growing Up Today Study, and U.S. Department of Agriculture food security scales measured nine social domains.	Participates completed online tool to identify problems, and then referral portion matched selected problem/needs to a list of agencies based on distance to participant's home.	The Online Advocate is a self-administered, web-based tool for SDOH screening and referral)	Process: + Identified income security, nutrition/fitness, and health care access as most frequent domains. The majority of youth choose to receive help. Nearly half had contacted a referral agency for their top priority. SDOH: + Change in complete or partial resolution of priority problem. Health: None reported Cost/utilization: None reported
Haas et al. (2015)^24^	Study design: Prospective RCT Sample: 707 low-SES adult smokers who are Black, Hispanic, or White (399 in intervention group, 308 in control group) Quality: low	Primary care practices	Used ArcMap 10.0 (Esri) to geocode participants' HER mailing addresses to append median household income estimates based on 2010 census tract as a proxy for socioeconomic status	Use EHR to identify eligible participants and then used interactive voice response to recruit.	HelpSteps.com (a web-based referral system to help users select referrals to local health and human service agencies categorized into 13 social resource domains	Process: 46.7% requested a HelpSteps.com referral; and 20.1% reported using this referral. SDOH: Community resources for physical activity, educational opportunities, and job counseling were the most common referrals requested. Health: Individuals who reported using their referral for the community resource were much more likely to quit than those who did not. The only intervention component associated with quitting was use of a community referral. Cost/utilization: None reported
Sege et al. (2015)^25^	Study design: RCT Sample: Parents of newborns <10 weeks of age at pediatric primary care clinic; 330 families (163 control and 167 intervention) Quality: Moderate	A major urban teaching hospital	Items concerning family hardship were adapted from the Fragile Families study. Food, housing, Medical/legal, income, utilities, and others	Assessments, conducted in English or Spanish, were administered at well-child visit. Surveys were administered in our general clinical research center by trained research staff. All cases were discussed in weekly case conferences that included the Healthy Steps Director, Medical/Legal, staff member, and a primary care pediatrician.	Project DULCE plus FS in infants' health care setting. Intervention group was paired with trained FS who provided support (home visit) and direct assistance access resources.	Process: most respondents (73%) reported at least one type of hardship; 61% food insecurity, and 45% housing insecurity, 42% struggle to pay utility bills. SDOH: + Contact with FS, increase access to resources. Health: None reported Cost/utilization: + More likely to complete immunization, have routine preventive care visits, and less likely to visit ER.
Bronstein et al. (2015)^26^	Study design: RCT Sample: 89 patients ≥50 years old and have moderate-to-high risk of readmission postdischarge (45 intervention, 44 standard care control) Quality: Moderate	Reginal hospital	No screening tool/process was mentioned. Financial constraints, lack of knowledge about the role of their PCP, accessing and taking prescribed medications, and accessing transportation.	MSW intern met with identified at risk patients for recruitment and consent.	Social work-led care coordination intervention. MSW intern assisted patients to assess, identify, and alleviate barriers at home after discharge.	Process: + Patients' response to intervention. SDOH: None reported Health: None reported Cost/utilization: + Decreased 30-day hospital readmission rate.
Tomita and Herman (2015)^27^	Study design: RCT Sample: 150 severely mentally ill, previously homeless adults discharged from inpatients psychiatric care (77 intervention, 73 control) Quality: High	Inpatient care	Homelessness No screening tool/process was mentioned.	Patients be referred to community-based services plus receive CTI delivered by trained social services workers, supervised by clinical research staff	CTI, a time-limited care coordination model to strengthen community support network. It was designed to prevent homelessness and other adverse outcomes during the period after discharge from inpatient psychiatric treatment.	Process: No significant differences in continuity of care process measures (perceived ease of access, stability of patient/provider relationship, severity of instability patient/provider relationship) SDOH: None reported Health: None reported Cost/utilization: None reported
Fox et al. (2016)^28^	Study design: pre/post pilot intervention Sample: 116 patients Quality: very low	A pediatric weight management clinic	Screened for food insecurity using a validated two-item instrument (Hager et al., 2010) completed by the parent/guardian.	Patients be referred to Second Harvest Heartland for SNAP enrollment assistance.	Second Harvest Heartland (nation's largest food banks) outreach workers provided direct assistance with the SNAP enrollment process to the families of patients attending the pediatric weight management clinic.	Process: 28 (24%) endorsed food insecurity, and 40 (34%) were eligible for SNAP enrollment assistance. Even when given direct access to SNAP enrollment assistance, only a small minority of patients (8%) completed the SNAP enrollment process. SDOH: Not reported. Health: No difference in BMIs between patients with and without food insecurity. Cost/utilization: None reported
Gottlieb et al. (2016)^29^	Study design: RCT Sample: 1054 parents in total (553 in the active control arm, and 501 in the navigation intervention arm). Quality: Moderate	Pediatric primary and urgent care clinics in two safety-net hospitals	A standardized 14-item social and mental health needs screening questionnaire: housing stability and habitability, food and income security, childcare and transportation needs, employment, legal concerns, medical insurance and other public benefit enrollment, and concerns about adult house member's mental health.	Patient navigators administered a baseline survey on family social risk factors.	Navigator Intervention. Caregivers were offered a meeting with the navigator immediately after the child's clinic visit or by telephone later. Navigators provided targeted information related to community, hospital, or government resources addressing needs that participants had prioritized.	Process: None reported SDOH: Social needs decreased by self-report. Health: Caregivers reported significantly greater improvement in their child's health. Cost/utilization: None reported
Morales et al. (2016)^30^	Study design: Retrospective observational cohort with propensity score-matched design. Sample: 145 female patients seen in the obstetrics clinic Quality: Moderate	Obstetrics clinic in a CHC	Food insecurity	(1) Screening using a standardized assessment form at visit check-in or by referral from a provider if food insecurity was uncovered during the course of a visit; (2) after SNAP or WIC enrollment, Patients were assisted with obtaining food resources, or provision of information regarding local food pantries.	Food For Families intervention—identifies food-insecure patients and connects them with food resources, such as the SNAP, the Special Supplemental Nutrition Program for WIC, and food pantries.	Process: 67% (97 of 145) of referred women enrolled. SDOH: None reported Health: During pregnancy, women who were referred to and enrolled had a better SBP and DBP than those who were not referred. Women not referred to Food for Families and those who were referred but did not enroll experienced a rise in BP during pregnancy No difference in blood glucose. Cost/utilization: None reported
Juillard et al. (2016)^31^	Study design: Longitudinal observational analysis of prospectively collected program and trauma registry data (2005–2014) Sample: 459 violently injured youth and adults (10–35 yrs). Quality: Low	Level I Trauma center	Violence, high risk of reinjury	Patients were identified through hospital records within 24 h (weekdays)/48 h (weekends) of presenting to the ED. Bedside risk assessment to identify individuals who are at high risk of reinjury.	The Wraparound Project, hospital-based case management VIP-intensive, culturally competent case management (mentorship, advocacy, and shepherding to community resources).	Process: + Identification of social needs; Mental health services, victim-of-crime compensation, employment, and housing were the most frequently identified needs. SDOH: Successfully met victim of crime compensation and visa needs. Low success in meeting employment needs and attaining driver's license. Health: Violent reinjury rate decreased compared with a historical control. VIP prioritization of housing needs may reduce future reinjury. Cost/utilization: None reported
Nguyen et al. (2016)^32^	Study design: Retrospective observational, pre/post intervention, pilot. Sample: 18 self-identify as Hispanic with diabetes (ages 60 or older) Quality: Very low	A university-affiliated, FQHC	Use a checklist for (1) housing, (2) transportation, (3) food, (4) clothing, (5) dental and prescription services, (6) employment, or (7) family and social services. Volunteers used a public, online database of local nonprofit organizations (www.211oc.org) to search for relevant referrals to free or low-cost community programs	Health Connectors volunteers were premedical undergraduate students or college graduates. Volunteers were trained by clinic personnel. During continuity clinic hours, volunteers worked in coordination with medical assistants, to administer a brief interview during patients “wait time” to administer screening and assistance locating or accessing local resources.	The Intervention: Health Connectors. Health Connectors volunteers work with patients to identify needs and locate resources to meet those needs.	Process: The most common requests were for low-cost dental clinics, food assistance, and housing support. The rate of referral uptake (50%) is high. SDOH: None reported Health: No significant changes in diabetes care and self-efficacy were detected. Cost/utilization: None reported
Berkowitz et al. (2017)^33^	Study design: difference-in-difference evaluation. Sample: 5125 adults patients screened between October 2012 to September 2015 (1774 health leads; 3351 comparison) Quality: Moderate	Academic primary care practices.	A standardized screening form that allows patient to self-identify unmet resource needs related to food, medication, transportation, utilities, employment, elder care services, and housing.	Health Leads consists of screening for unmet needs at clinic visits and offering those who screen positive to meet with an advocate to help obtain resources or receive brief information provision.	The Health Leads program, social needs screening, and referral physicians “prescribe” social resource and Health Leads volunteers assist patients by providing relevant community resources.	Process: + Identification of social needs; + change in connections to and enrollment in community programs. SDOH: None reported. Health: + Change in improvement of SBP and DBP, LDL-C level. No HbA1c change. Cost/utilization: None reported
Cohen et al. (2017)^34^	Study design: quasi-experimental trial; pre-, post-intervention Sample: 177 SNAP enrolled adults, primary care patients. Quality: Low	An academic outpatient family medicine and pediatrics practice serving a low-income, racially and ethnically diverse community.	Food insecurity	In the waiting room, study staff provided participants a brief verbal explanation of DUFB. Participants were given print copies of DUFB, local farmers market maps, and a one-time $10 voucher.	Brief clinic-based intervention associated with increase in uptake of SNAP incentive program-DUFB	Process: 59% eligible adults enrolled. SDOH: Fourfold increase in uptake of a SNAP incentive. Health: Behavior change+fruit and vegetable consumption increased. Cost/utilization: None reported
Allen et al. (2017)^35^	Study design: RCT Sample: 399 Latino adult patients with poorly controlled T2DM A1C >7.5% (58 mmol/mol) seen at urban safety-net clinics with known high social needs. Quality: high	Clinics within two FQHCs	A brief, computerized, EHR-linked patient assessment tool (Internet-based) embedded within a diabetes team care Dashboard 20-item screening tool measures social distress (family needs and problems, the lack of money for basic living expenses, family conflicts, legal problems, overcrowding, living in an unsafe neighborhood, physical or mental abuse, discrimination, and job loss or underemployment)	Patients surveyed by telephone and in person when using a culturally sensitive survey strategy.	Intervention involved one-on-one diabetes education tailored to each patient's individual clinical, behavioral, and social distress profile and referred each person to local services as needed for social distress issues. It was delivered by bicultural, bilingual diabetes educators (CDEs).	Process: >90% participants reported at least one social distress issue and 11 social distress items (out of 20) were present by ≥30% of patients. SDOH: Social distress score declined. Health: None reported Cost/utilization: None reported
Patel et al. (2018)^36^	Study design: pre/post one-group design pilot study Sample: 104 adults with diabetes Quality: low	Endocrinology clinic	Financial burden	Research staff approached patients in the clinic waiting room for financial burden through seven survey questions adapted from CDC surveys.	Financial burden resource tool, which provided tailored, low-cost resource options for diabetes management and other social needs.	Process: Patients reported the tool highly acceptable. + Improvements in discussion of cost concerns with nurses and pharmacists. SDOH: None reported Health: None reported Cost/utilization: None reported
Hsu et al. (2018)^37^	Study design: Non-RCT (Mixed-Methods Approach) Sample: adult patients Intervention CRS (*n*=420) vs. matched controls (*n*=1045) Quality: Moderate	A large health plan	Multiple SDOH Screened with patient-Centered Medical Home Items, the Patient Activation Measure, physical activity, and social isolation questions adapted from the Behavioral Risk Factor Surveillance System, a single-item health status question, questions about goal setting, action, planning.	Patients can be referred to CRS and automatically enter into an EHR-based CRS registry.	CRS referral, and follow-up. CRS role focuses on three key activities: (1) directly help with patients to access community resources and set health-related goals, (2) researching and becoming familiar with community resources, and (3) increasing the primary care team's knowledge of those resources.	Process: Patients were satisfied with services of CRS, + increased number of face-to-face primary care visits, and secure message use, and secured message threads. SDOH: None reported Health: No significant impact on behavioral health. Cost/utilization: No significant impact on total overall primary care utilization, emergency department, urgent care, outpatient, nurse visits, and telephone use.
Kangovi et al. (2018, 2020)^38,39^	Study design: parallel-group, multisite RCT Sample: 592 adult patients with multiple chronic diseases, 304 in the intervention group and 288 in the control group. Quality: High	Multiple sites, including primary care clinics and FQHC	Multiple SDOH, including trauma, food insecurity, housing instability, drug and alcohol use, or family stress.	CHWs used a semistructured interview guide to get to know the patients holistically and assess their socioeconomic determinants of health. IMPaCT was highly standardized in terms of its approach to hiring, training, work-flows, supervision, documentation, and intervention fidelity.	IMPaCT intervention, in which CHWs provide tailored social support, navigation, and advocacy to low-income patients achieve health goals.	Process: Almost half-delayed health needs, about 40% lack of basic needs; +improvement in patient activation; +more likely to report the highest quality of care SDOH: None reported Health: + Improvement in self-rated physical health and disease self-management. No changes in HbA1c, BMI, CPD, and SBP Cost/utilization: Fewer days in length of stay and hospitalization; less likely to have repeat hospitalization, including 30-day readmission; every dollar invested in the intervention return $2.47 to an average Medicaid payer within the fiscal year.
Barton et al. (2019)^40^	Study design: Quality improvement and mixed methods study (pre/post chart review) Sample: 48 nontraditional high school students. Quality: Low	A school-based clinic in a nontraditional high school in an impoverished area.	Multiple social needs (depression, anxiety, tobacco, alcohol, and drug use, interpersonal violence, food insecurity, housing insecurity, health literacy, nutrition, physical activity, and risky sexual behaviors). Screen tool: SBDOHs screening bundle	School clinical staff: the nurse practitioner interviewed the students and record their answers into REDCap, a secure online database was used to store screening results in lieu of the clinical EMR whose installation was pending.	School-based clinic brief intervention. Any student having a positive response to one or more questions on the screening set was further interviewed by the nurse practitioner before any final referral were made.	Process: 48 students completed the screening set. SDOH: Identified of adverse SBDOHs increased using pre and post-chart review: + in behavioral health referrals, + in nutritional counseling referrals, + in the number of social services referrals (including gynecology-related referrals for birth control). Health: None reported Cost/utilization: None reported
Buitron de la Vega et al. (2019)^41^	Study design: observational, feasibility study Sample: 1522 patients Quality: Low	Urban safety net hospital	Multiple domains of SDOH: homelessness, housing insecurity, food insecurity, inability to afford medications, lack of transportation, utilities, caregiving, unemployment, and educational aspirations.	Screen for SDOH, capture responses as standard ICD-10 codes in the EHR, and provide patients with resource referral guides to help address unmet social needs.	THRIVE, an SDOH screening and referral program, in General Internal Medicine Clinics THRIVE was modeled after pediatrics WE CARE, a SDOH screening and referral program	Process: Employment, food insecurity, and problems affording medications were the most prevalent concerns; 82% patients with ≥1 identified needs (excluding education) had the appropriate ICD-10 codes added to their visit diagnoses; 86% patients who requested resources received a relevant resource referral guide. SDOH: None reported Health: None reported Cost/utilization: None reported
Schickedanz et al. (2019)^42^	Study design: A prospective, quasi-experimental study using an intent-to-treat propensity-weighted difference-in-differences analysis Sample: 34,225 adult patients (7107 intervention, 27,118 control) Quality: Moderate	A large integrated health care delivery system (KPSC)	Multiple domains of SDOH Health Leads staff call patients with a 14-question social needs screener, which took 5–7 min to complete.	KPSC worked in partnership with Health Leads, a nonprofit organization for screening and navigation.	A telephonic social needs screening and social needs navigation and referral.	Process: Most (53%) patients screened reported social needs. The most common social needs reported were financial strain and food insecurity. SDOH: only a minority (10%) of those with a need were able to connect with resources to address these needs Health: None reported Cost/utilization: total utilization (a count visits in ER, outpatient and/or inpatient) decreased
Finkelstein et al. (2020)^43^	Study design: RCT Sample: 800 adult patients (399 intervention, 401 control) Quality: high	Several regional hospitals	Multiple domains of SDOH—complex social needs, including difficulty access social services, lack of social support, coexisting mental health condition, active drug habit, and homelessness.	The Camden Coalition of Health care Providers (the Coalition) uses real-time data on hospital admissions to identify superutilizer patients, an approach referred to as “Hotspotting.” Their program—the Camden Core Model—is a time-limited, intensive care transition program that targets superutilizer patients.	Health Care Hotspotting Intervention, is the Camden Coalition of Health care Providers’ Care Management Program. The program targets “superutilizers” of the health care System—specifically adults with two or more hospitalizations in the last 6 months and two or more Chronic conditions—with intensive care-management services in the 1–3 months following hospital discharge.	Process: Engagement with the program was high (95%); a home visit and a visit to a provider's office after discharge—were achieved <30% of the time due to patients' lack of stable housing or a telephone and their behavioral health complexities and providers' few available appointments. SDOH: No change in participation in government benefits or assistance—rates were low. Health: None reported Cost/utilization: No significant effect on participants' 180-day readmission rate
Gottlieb et al. (2020)^44^	Study design: RCT Sample: 661 caregiver/child dyads in total (302 dyads in the written resources group, and 309 in the in-person assistance group). Quality: high	A pediatric urgent care clinic in a large urban, safety-net hospital	18-item social risk screening questionnaire included housing instability and habitability, food and income security, childcare and transportation needs, employment, legal concerns, medical insurance, and other public benefits enrollment.	A patient navigator administered a brief social risk survey.	Compare two interventions: one with written resources, and the other with in-person assistance	Process: None reported SDOH: Caregivers who received written resources alone reported fewer risks while those receiving written resources plus in-person assistance reported fewer risks (both *p*<0.001). Health: Small but statistically significant improvement in child health for both written resources and written resources plus in-person assistance; both *p*<0.001. Cost/utilization: None reported
Poleshuck et al. (2020)^45^	Study design: RCT Sample: 225 adult women with elevated depressive symptoms, ESR intervention 112, PSP intervention 113. Quality: high	Women's health clinics serving primarily Medicaid-eligible patients	Multiple domains, including stable housing, food scarcity, clothing, transportation, and legal needs. Use a social needs questionnaire to assess.	Research assistants screened for depression in the waiting rooms of women's health clinics	Compare two interventions: PSP or ESR. PSP was CHW intervention tailored to women's priorities and social context, and the ESR was a lower intensity intervention providing a personalized resource list and modest social support.	Process: most (75.3%) reported ≥1 social needs. Most common needs were insufficient clothing, food scarcity, and lack of transportation. Both groups were satisfied with two interventions. SDOH: None reported. Health: + Improvement for depression in both groups and improvement in QOL in PSP vs. ESR. Cost/utilization: None reported
Wallace et al. (2020)^46^	Study design: Mixed-methods feasibility trial, pilot Sample: 210 patients (*n*=162 with complete data that could be linked (social needs, details of 2-1-1 encounters and referrals, and health service usage data from Epic). Quality: low	A large academic center emergency department	Multiple social needs: housing and utilities; food assistance; transportation needs; legal resources; mental health and addiction services; medical, dental, and vision insurance; employment services; education and training; and domestic violence and abuse.	Screening was completed by five ER department registration staff early in the admission process. Electronic referring patients to the 2-1-1 community-based call center. Results of the 2-1-1 follow-up contact were extracted from 2-1-1 information system and by using the unique ID, linked with initial screening results and select fields extracted from Epic's data warehouse	An academic/community partnership for 2-1-1 referral to facilitate access to community resources and follow-up	Process: 61% of patients reported one or more need; among those who wanted referral, 49% were ultimately reached by 2-1-1, which provided an average of four community referrals. SDOH: None reported Health: None reported Cost/utilization: Increased ER visits with patients with social needs; There was no differences in hospitalizations; patients with at least one social needs had a significant increase in ER use while patients with no needs had an increase in primary care visit.
Pantell et al. (2020)^47^	Study design: RCT Sample: 1300 in total (663 in the written resources group and 637 in the patient navigation group). Quality: high	Primary and urgent care clinics of two safety-net hospitals in northern California.	Multiple SDOH domains. Use a questionnaire to collect data on multiple household social risk: food insecurity, problem paying utility, problem finding employment, housing instability, living in an unhealthy environment, other housing concerns, problem paying medical bills, lack of health insurance, etc.	Patient navigators administered a baseline survey on family social risk factors.	Caregivers met with a patient navigator to address family social needs.	Process: None reported SDOH: None reported Health: None reported Cost/utilization: Children decreased risk of hospitalization within 12 months; no difference in risk of an emergency department visit
Henschen et al. (2021)^49^	Study design: Pragmatic RCT Sample: 151 patients in total (75 in the intervention group and 76 in the control group). Quality: high	Urban academic hospital in Chicago, IL	Multiple SDOH domains. In-depth psychosocial assessment to identify needs such as housing, behavioral health, access to food and medication, home care, and transportation.	CHAMP team members created and maintained the comprehensive care plan, coordinated follow-up care, and connected patients to existing community resources.	The CHAMP. Components include helping patients set health-related goals, develop care plan that was accessible to health system connect patients to existing community resources.	Process: None reported SDOH: None reported Health: None reported Cost/utilization: No difference in outcomes, intervention group was associated with higher 30-day inpatient readmissions 180 days following enrollment compared with a control group.
Ibe et al. (2021)^48^	Study design: Cluster-RCT Sample: 1890 adults (from 30 participating practices) with uncontrolled hypertension and at least one other cardiovascular disease risk factor Quality: high	Primary care practices across five health systems in Maryland and Pennsylvania	Multiple SDOH domains. Used the SDOH framework to guide the identification of measures across four of the six domains articulated by Artiga and Hinton: economic stability, education, food, and community and social context.	NCM-led care team members assess patient's physical and psychosocial health, daily functioning, and social circumstances. After identifying the social needs that requires immediate attention (e.g., food insecurity, domestic violence, and poor or unstable housing), NCM refers patients to receive help from a CHW. Once in contact with a patient, the CHW connects the patient to community resources, and uses patient-centered communication to encourage adherence to self-management goals.	The RICH LIFE Project comparing the effectiveness of enhanced standard of care, SCP, to a multilevel intervention, CC/SC, for improving BP control and patient activation and reducing disparities in BP control.	Process: Patients who were unable to work and those with higher health literacy were less likely to engage with the collaborative care team than those who were employed full time or had lower health literacy, respectively. Patients had a greater likelihood of being referred to a CHW by their care manager if they reported higher health literacy, perceived stress, or food insecurity, while those reporting higher numeracy had lower odds of receiving a CHW referral. SDOH: None reported Health: None reported Cost/utilization: None reported

BMI, body mass index; BP, blood pressure; CC/SC, collaborative care/stepped care; CDC, Centers for Disease Control and Prevention; CDE, Certified Diabetes Educator; CHAMP, Complex High Admission Management Program; CHCs, community health centers; CHWs, community health workers; CPD, cigarettes per day; CRS, community resource specialist; CTI, critical time intervention; DBP, diastolic blood pressure; DUFB, Double Up Food Bucks; DULCE, Developmental Understanding and Legal Collaboration for Everyone; EHR, electronic health record; ER, Emergency Room; FQHCs, Federally Qualified Health Centers; FS, family specialist; ICD-10, *International Classification of Diseases, 10th Revision*; IMPaCT, Individualized Management for Patient-Centered Targets; KPSC, Kaiser Permanente Southern California; LDL-C, low-density lipoprotein cholesterol; MSW, Master of Social Work; NCM, Nurse Care Managers; PCP, Primary Care Provider; RCT, randomized controlled trials; SBDOHs, social and behavioral determinants of health; SBP, systolic blood pressure; SCP, standard of care plus; SDOH, social determinants of health; SNAP, Supplemental Nutrition Assistance Program; T2DM, type 2 diabetes mellitus; VIP, violence intervention program; WIC, women, infants, and children.

### Study design, sample characteristics, quality of studies, and clinical settings

Out of 28 studies reviewed, 14 were randomized controlled trials (RCT)^22,24–27,29,35,38,39,43–45,47–49^ and the other study designs included quasi-experimental design or pre/post designs. Samples included adults and parents/caregivers of pediatric patients. In terms of clinical settings, the majority of the studies were conducted at health care organizations servicing low-income individuals and families, such as Federally Qualified Health Centers (FQHCs), safety-net hospitals, community health centers, and primary care practices where the prevalence of social and economic barriers to health is high among the patient population. The quality ratings of studies varied from very low to high based on the GRADE approach.^21^

### SDOH-EHR integration (i.e., screening, data collection, integration into EHR) into the clinical workflow

All studies provided some basic information regarding the screening for SDOH and integration of such screening into the workflow. The integration process was performed using any secure electronic device (e.g., tablets, computers) and software (e.g., electronic data capture systems/tools such as REDCap). The collected data were further integrated into EHR systems (i.e., Epic) of clinical institutions to identify at-risk individuals and to guide targeted intervention strategies or referral services to address corresponding unmet social needs in follow-up appointments. Studies reported either using EHR to identify at-risk patients who may need social and economic assistance,^24,31,43^ or in-person screening—usually including descriptions about responsible care team members and the time frame for SDOH screening.^22,25,26,29–34,36,38–40,44,48,49^ However, the time frame for data entry into the EHR was not discussed in studies. Of all the studies reviewed, only one^41^ reported the process of SDOH screening and capturing responses as standard *International Classification of Diseases, 10th Revision* (ICD-10) codes in the EHR.

### Target SDOH and interventions

The majority of the studies targeted and addressed multiple SDOH domains, including housing instability and inhabitability (i.e., supportive housing, rent subsidies, assist in household heat, or paying utility bills), basic needs (i.e., provide shoes, clothing), food insecurity, transportation, low income/financial constraints, unemployment/loss of job or underemployment, childcare, medical/legal assistance, lack of medical insurance and other public benefits enrollment, and concerns about adult household member's mental health, drug and alcohol use, neighborhood safety, trauma, exposure to violence (including domestic violence), and discrimination. Frequently, studies described participants having two or more social needs. The most often reported social needs were related to housing, food, childcare, and finances.

Interventions all targeted individual-level SDOH, including social and economic needs. These interventions vary by the level of intensity of their approaches. The simplest approach involved health system staff identifying social risks and needs among patients and following up either by distributing a booklet of community resources or referring patients to community resources or social services. This practice usually had little follow-up or process evaluation to determine whether patients received the resources they needed. The second and more advanced approach involved a patient navigation process (usually administered by patient navigators, social workers, or case managers) that helped navigate patients to external human or social services based on EHR-embedded SDOH information. The third approach involved a transition care coordination model that helped patients move through different levels and types of care at different facilities. Referrals, developing an individual plan for coordination of care, and managing information exchange between providers and other social service organizations is the responsibility of the care coordinator. This approach usually targets patients at higher risk for hospital readmission.^26,27,43,49^

### Outcome measures

Based on the Centers for Disease Control and Prevention program evaluation framework,^50,51^ we organized the outcome measures into four categories, including (1) process measures, (2) short-term outcome directly related to social needs, (3) intermediate outcome or impact on health outcomes, and (4) long-term outcome or impact on health care cost or utilization. Process measures established whether program activities were being implemented as proposed. Meanwhile, outcome measures evaluated effectiveness of the program on the short-term outcomes on the targeted population, for example, changes on the SDOH, intermediate outcomes such as the health outcome, and the long-term outcome, such as the impact on the health care cost and utilization. Most studies (*n*=23, 82%) reported the findings related to the process measures, and nearly half (*n*=12, 43%) reported outcomes related to SDOH. By contrast, 39% (*n*=11) of the studies reported health outcomes, and only 32% of the studies (*n*=9) presented findings related to the impact on health care cost and utilization.

#### The process measures and impacts on patients' social needs

The process measures include number of social referrals; number of people able to identify their social needs; rate of patients who agreed to receive help (referral uptake); percentage of patients who contacted a referral agency, enrolled in new community resources, reported using community resources or social services; and patients' overall satisfaction with the referral/patient navigation services or satisfaction with care.^22–25,28,30–41,43,45,46^ Findings were largely positive for process measures. Similarly, although fewer studies reported findings related to a program's impact on patients' social needs change, those studies led to improved access to resources, resolutions of identified problems, or decreased self-reported social distress score.^22,23,25,29,31,34,35,40,42,44^

#### The health outcomes

Overall, few studies included the health measures. The health outcomes measures had little consistency across studies and varied by population age (adults vs. the pediatric population). Health outcome measures included health-related behaviors^24,34,37^ (e.g., quit cigarette smoking, fruit and vegetable consumption, etc.), physical health,^28,30,32,33,38,39,44^ quality of life and depression,^45^ and injury.^31^ The evidence of program impacts on health is mixed. Some studies reported improved health, including increased likelihood of quitting smoking,^24^ improvements in child health (caregiver self-report),^29,44^ better blood pressure during pregnancy,^30^ decreased violent reinjury rate,^31^ improvements in systolic blood pressure (SBP) and diastolic blood pressure, lowered low-density lipoprotein cholesterol level,^33^ increased fruit and vegetable consumption,^34^ self-rated physical health and disease self-management,^38^ improvements in quality of life, and reduction of reported depression.^45^ In contrast, others reported no changes in behavioral health,^37^ in patient-selected chronic disease (HbA1c, body mass index, SBP, or cigarettes per day),^38,39^ or hemoglobin A1c.^33^

#### Health care cost and utilization

Across studies, health care cost and utilization measures varied widely while findings were mixed but trending positive. Several studies reported positive impacts on health care cost and utilization measures, such as improved immunization completion rates, routine visits for preventative care, lowered ED visits/hospitalizations among children^25,47^; decreased 30-day hospital readmission rates, repeat hospitalization, or total utilization in adults,^26,38,39,42^ while others reported null findings in adults.^37,43,46^ Only one study reported the cost-effective findings regarding return in investment in an average Medicaid payer within the fiscal year.^42,43^

## Discussion

This systematic review of 28 studies conducted within the U.S. health system examined and synthesized the process of integrating SDOH screening into EHRs and the impact of social needs intervention on a full range of outcomes. In studies that reported the process measures, findings were generally positive. Similarly, findings related to programs' effects on patients' SDOH-related social needs change demonstrated improved access to resources. However, these interventions impact on health outcomes and health care cost and utilization were mixed. As a whole, this study contributed to the literature by shedding light on several clinical and population health-related research questions regarding the implementation and effectiveness of social needs screening and interventions.

First, our study expands the literature on the current clinical workflow for SDOH screening and integration in EHRs. To our knowledge, few review articles provided clear descriptions of the clinical workflow involved in screening for and integrating SDOH in EHRs, and the subsequent interventions through social prescriptions, such as health professionals working with social workers or community connectors to connect patients who have social needs with community resources or social services. Our findings indicate that research is needed to explore more efficient means of SDOH screening/EHR integration (i.e., screening, data collection, and integration into EHR) into the clinical workflow. Studies reviewed in this article reported various methods for screening and coding, and only one study explicitly described using codes from *International Classification of Diseases, 10th Revision, Clinical Modification* (ICD-10-CM) to document patients' SDOH. To facilitate the integration into the clinical workflow, the essential first step is data standardization.

It is critical to implement data standardization in clinical decisions to ensure suitable interventions and referral practices are deployed by nurses, physicians, and health staff to address SDOH and the sharing of social needs data across health care facilities.^52,53^ In addition, data standardization is important for valid data aggregation across EHR system of various practices and communities. However, no uniform, accepted data model or established criteria represent these determinants in EHR systems.^53,54^ Similar to utilizing ICD-10-CM codes and Current Procedural Terminology in diagnosis and procedures/billing, respectively, the American Hospital Association has suggested documenting and coding SDOH using the many available ICD-10-CM codes.^55^ For example, categories Z55–Z65 (“Z codes”) of the ICD-10-CM codes are available for hospitals to capture patient's social needs. Current Z codes include data on patient's socioeconomic status such as access to employment, education and literacy, housing, or adequate food and/or water.

Although the American Hospital Association wants to promote the usage of Z codes, health systems' adoption of SDOH Z codes remain slow. Based on a retrospective cohort study using the 2016 and 2017 National Inpatient Sample from the Healthcare Cost and Utilization Project database—the largest publicly available all-payer inpatient care database in the United States, of 14,289,644 admissions, 269,929 (1.9%) had an associated SDOH Z-score content.^56^ This number indicates SDOH Z-scores are not being utilized to an appropriate degree within the health system. Thus, the coding being used at this time is probably reflecting the true burden of social needs poorly for hospitalized patients as unstable housing was reported in 37% of patients with diabetes in a national survey of safety net patients,^57^ which is much higher than 1.9%.

In addition, particularly in light of the COVID-19 pandemic, we have observed how SDOH pathways, when linked with pre-existing comorbidities, apply a burden of morbidity and mortality that is much higher than expected upon minority communities.^58–60^ Without accurately and timely collecting, documenting, identifying, and addressing SDOH, we cannot effectively improve health outcomes and reduce the health disparities that have long existed to realize equity in health care.

Second, consistent with previous review articles, our review showed that current studies that included both health and health care cost and utilization measures were limited in number and mixed in their results to provide conclusive evidence for the effectiveness of reducing cost/utilization and improving clinical outcomes.^14,17,61^ In the current review, about half of all studies used RCT design, and only one study reported cost-effectiveness.^42,43^ More rigorously designed studies are needed as the field moves forward to clarify cost-effective intervention approaches. The sample sizes of studies vary from 18 to 34,225 in nonexperimental design studies and from 89 to 1300 in experimental design studies.

The measurements vary widely even within the same outcome domains and thus make it difficult to compare and synthesize. For example, a study reported the total health care utilization (a count of all visits to the emergency department, outpatient, and/or inpatient) would limit our ability to compare the results in each usage. Null findings from studies that focused on participants with a high-risk or high-utilization rate may experience a widespread statistical phenomenon–regression to the mean,^62^ as seen in a recent RCT study—Camden Coalition of Healthcare “Hotspotting” intervention.^43^

Third, although interventions vary by the level of intensity, there was insufficient evidence that the intervention intensity and the health or cost and utilization outcomes were dose–response correlated. More contacts or heavy intensities in social needs interventions did not necessarily lead to better health outcomes. A recent randomized control trial compared two interventions in pediatric urgent care settings and suggested no statistically significant differences in the delivery of personalized printed resources about social services with or without longitudinal, in-person navigation services.^44^

Fourth, when examining the barriers to/facilitators of, and resources needed to implement effective social needs interventions in clinical settings, we must consider not only patients' uptake but also social service agencies' capacity. Research may assume that current social service agencies have the ability to address the social needs of all community members.^63^ But in reality, social service agencies have capacities that vary broadly by the type of social need (easier to manage food-related needs than housing needs or public transportation) and location of the community.^63^ This was documented in a recent study, which found that 2-1-1 referrals for food-related needs were the most likely to be met, while referrals for housing-related needs were the least likely.^64^ It is not surprising that studies found that only a small portion (10%) of those with social needs could connect with resources to address these needs^42^ or low participation rates in government benefits or assistance.^43^

A recent review^16^ suggested a deeper understanding of an individual's social needs patterns, timing, and sequences. Our findings suggest that for social needs to be met there must be a shift from reactive damage control to proactivity that seeks to address many upstream determinants (such as racism) at the population level.

Fifth, we call for future studies to report the capacity for innovation and the implementation of value-based payment models. The value-based payment has the potential to spur innovation in upstream prevention, such as screening patients for social risks. By doing so, the health system (i.e., physician practices) can take the early step of addressing social needs that lead to poor health. None of the social screening and EHR integration studies reviewed in this study reported the implementation of a value-based payment model.

This finding was not surprising, although, as a recent cross-sectional study of social risk screening by U.S. physicians indicated that implementing social risk screening in the U.S. health care setting was not associated with the practices' overall exposure to value-based payment.^65^ Instead, Brewster et al. found that adopting social risk screening in the U.S. health care setting was associated with high innovation capacity and focusing on low-income populations, regardless of payment incentives.^65^ Most of the study settings reviewed in our study were at health care organizations servicing low-income individuals and families with limited innovative capacities, such as FQHCs, safety-net hospitals, and community health centers.

The recent finding by Brewster et al. and our observations have important implications. First, the current practice capacity for innovation, rather than payment incentives, is the primary driver of social risk screening. Second, there is an urgent need to standardize approaches and implementation assistance to reduce the level of innovative capacity required to introduce social risk screening at the practice level.

Lastly, one solution U.S. health systems can provide to improve community capacity is to make more direct investments instead of just screening and making referrals. For example, a recent study^66^ identified that 78 new programs (involving 57 health systems, including 917 hospitals) made direct financial investments (at least $2.5 billion) in SDOH from 2017 to 2019. Those investments were committed to direct SDOH, including housing, employment, education, food security, social community context, and transportation. We applaud the efforts of these health systems and encourage them to continue direct and large investments in SDOH.

### Limitations

This systematic review has at least two limitations. First, this review has strict inclusion and exclusion criteria, and all studies reviewed were limited to those in the U.S. health system. In addition, the majority of the studies were performed in community health centers. Therefore, we caution against generalizing our findings to other settings. Second, there may be review-level limitations such as incomplete retrieval of identified research and the study/outcome-level limitations, including heterogeneous nature of the studies (variety of outcome measures, different study designs) and the small number of published studies with rigorous design. These limitations make it difficult to quantify the intervention's effects by systematically conducting a meta-analysis. We recommend carefully designed meta-analyses on outcomes related to effectiveness and health care cost and utilization in future studies with rigorous study designs.

## Conclusion

In conclusion, this systematic review found positive findings on the effects of interventions on process measures and patients' change on SDOH-related social needs. However, the findings were mixed about the impacts of these interventions on health and health care cost and utilization. Our review supports the current policy efforts to increase U.S. health systems' investment toward directly addressing SDOH, such as housing, food security, and job training. To effectively screen and respond to SDOH in health care settings, a number of changes must occur: federal standards must be established for reflecting SDOH data in EHR, data collection of SDOH, or social needs must be incentivized through financial or quality measures, and rigorous research that examines the impact of these actions must be expanded. While effective interventions can be more complex or resource intensive than an online referral, health care organizations hoping to achieve health equity and improve population health must commit the effort and investment required to achieve this goal.

## Supplementary Material

Supplemental data

## Abbreviations Used

BMI: body mass index
BP: blood pressure
CC/SC: collaborative care/stepped care
CDE: Certified Diabetes Educator
CHAMP: Complex High Admission Management Program
CHCs: community health centers
CHWs: community health workers
CPD: cigarettes per day
CRS: community resource specialist
CTI: critical time intervention
DBP: diastolic blood pressure
DUFB: Double Up Food Bucks
DULCE: Developmental Understanding and Legal Collaboration for Everyone
EHRs: electronic health records
ER: Emergency Room
FQHC: Federally Qualified Health Center
FS: family specialist
GRADE: Grading Recommendations Assessment Development and Evaluation
ICD-10-CM: *International Classification of Diseases, 10th Revision-Clinical Modification*
IMPaCT: Individualized Management for Patient-Centered Targets
KPSC: Kaiser Permanente Southern California
LDL-C: low-density lipoprotein cholesterol
MeSH: medical subject headings
MSW: Master of Social Work
NCM: Nurse Care Managers
PCP: Primary Care Provider
PICOS: participants, interventions, comparisons, outcomes, and study design
PRISMA: Preferred Reporting Items for Systematic Reviews and Meta-Analysis
RCT: randomized controlled trials
SBDOHs: social and behavioral determinants of health
SBP: systolic blood pressure
SCP: standard of care plus
SDOH: social determinants of health
SNAP: Supplemental Nutrition Assistance Program
T2DM: type 2 diabetes mellitus
VIP: violence intervention program
WIC: women, infants, and children
