# Supplementary material for: Effectiveness of Social Needs Screening and Interventions in Clinical Settings on Utilization, Cost, and Clinical Outcomes: A Systematic Review
Source: Health Equity. 2022 Jun 24;6(1):454–75. doi: 10.1089/heq.2022.0010 (PMC9257553; doi:10.1089/heq.2022.0010)
Supplement: Supplemental data [file Supp_DataS1.docx]

**Supplement 1: Database search strategies**

| **Database: Ovid MEDLINE(R)** | |
| --- | --- |
| 1 | exp Health Services/ or exp Ambulatory Care Facilities/ or (((community or neighborhood) adj2 health center*) or CHC or CHCs or (health* adj2 (clinic* or service*))).mp. |
| 2 | exp Primary health care/ or ((primary adj1 (healthcare or health care or care)) or ((patient-centered or patient centered or patient-focused or patient focused) adj1 care)).mp. |
| 3 | exp Hospital/ or (hospital* or ((inpatient* or in-patient* or outpatient* or out-patient*) adj3 (care or service*))).mp. |
| 4 | exp Ambulatory Care/ or (ambulatory care or urgent care or clinic visit* or safety-net or safety net or Federally Qualified Health Center or FQHC).mp. |
| 5 | exp Emergency Service, Hospital/ or (emergency department* or emergency room* or emergency ward*).mp. |
| 6 | or/1-5 |
| 7 | Social Determinants of Health/ or (social determinant* or SDOH or SDH).mp. or ((unmet or under-met or social or economic) adj1 need*).mp. |
| 8 | exp Educational Status/ or (education adj2 (status or achievement*)).mp. |
| 9 | Employment/ or unemployment/ or poverty/ or (employ* or unemploy* or career* or occupation* or poverty or income).mp. |
| 10 | exp Food supply/ or (food adj1 (suppl* or assistance or securit* or insecurit*)).mp. |
| 11 | exp housing/ or (housing or homeless* or rent*).mp. |
| 12 | exp Public Assistance/ or exp Social welfare/ or (public assistance or social welfare or social service* or medicare or Medicaid or childcare or child care* or day care* or daycare* or utility assistance or medical legal).mp. |
| 13 | exp Insurance, Health/ or health insurance.mp. |
| 14 | exp domestic violence/ or (domestic violence or family violence or (child adj2 abuse) or elder abuse or spous* abuse).mp. |
| 15 | exp transportation/ or (transport* or commute or commuting).mp. |
| 16 | exp Interpersonal relations/ or exp social isolation/ or (interpersonal safety or (social adj2 (interact* or relationship or isolat*))).mp. |
| 17 | or/7-16 |
| 18 | exp Electronic Health Records/ |
| 19 | (((electronic or medical or personal) adj2 (medical record* or health record*)) or EHR or EHRs or EMR or EMRs or PHR or PHRs).mp. |
| 20 | or/18-19 |
| 21 | Mass screening/ or screen*.mp. |
| 22 | 6 and 17 and 20 and 21 |
| 23 | limit 22 to (English language and yr="2015 -Current") |
|  | |
| **Database: Scopus** | |
| 1 | TITLE-ABS-KEY(((community OR neighborhood) W/2 "health center*") OR CHC OR CHCs OR (health* W/2 (clinic* OR service*))) OR ((primary W/1 (healthcare OR "health care" OR care)) OR ((patient-centered OR "patient centered" OR patient-focused OR "patient focused") W/1 care)) OR (hospital* OR ((inpatient* OR in-patient* OR outpatient* OR out-patient*) W/3 (care OR service*))) OR ("ambulatory care" OR "urgent care" OR "clinic visit*" OR safety-net OR "safety net" OR "Federally Qualified Health Center" OR FQHC) OR ("emergency department*" OR "emergency room*" OR "emergency ward*") |
| 2 | TITLE-ABS-KEY(("social determinant*" OR SDOH OR SDH) OR ((unmet OR under-met OR social OR economic) W/1 need*) OR (education W/2 (status OR achievement*)) OR (employ* OR unemploy* OR career* OR occupation* OR poverty OR income) OR (food W/1 (suppl* OR assistance OR securit* OR insecurit*)) OR (housing OR homeless* OR rent*) OR ("public assistance" OR "social welfare" OR "social service*" OR medicare OR Medicaid OR childcare OR "child care*" OR "day care*" OR daycare* OR "utility assistance" OR "medical legal") OR "health insurance" OR ("domestic violence" OR "family violence" OR (child W/2 abuse) OR "elder abuse" OR "spous* abuse") OR (transport* OR commute OR commuting) OR ("interpersonal safety" OR (social W/2 (interact* OR relationship OR isolat*)))) |
| 3 | TITLE-ABS-KEY((((electronic OR medical OR personal) W/2 ("medical record*" OR "health record*")) OR EHR OR EHRs OR EMR OR EMRs OR PHR OR PHRs)) |
| 4 | TITLE-ABS-KEY(screen*) |
| 5 | #1 AND #2 AND #3 AND #4 |
|  | Limited to English language, 2015 - present |
|  | |
| **Database: Web of Science Core Collection** | |
| 1 | TS=((((community OR neighborhood) NEAR/2 "health center*") OR CHC OR CHCs OR (health* NEAR/2 (clinic* OR service*))) OR ((primary NEAR/1 (healthcare OR "health care" OR care)) OR ((patient-centered OR "patient centered" OR patient-focused OR "patient focused") NEAR/1 care)) OR (hospital* OR ((inpatient* OR in-patient* OR outpatient* OR out-patient*) NEAR/3 (care OR service*))) OR ("ambulatory care" OR "urgent care" OR "clinic visit*" OR safety-net OR "safety net" OR "Federally Qualified Health Center" OR FQHC) OR ("emergency department*" OR "emergency room*" OR "emergency ward*")) |
| 2 | TS=(("social determinant*" OR SDOH OR SDH) OR ((unmet OR under-met OR social OR economic) NEAR/1 need*) OR (education NEAR/2 (status OR achievement*)) OR (employ* OR unemploy* OR career* OR occupation* OR poverty OR income) OR (food NEAR/1 (suppl* OR assistance OR securit* OR insecurit*)) OR (housing OR homeless* OR rent*) OR ("public assistance" OR "social welfare" OR "social service*" OR medicare OR Medicaid OR childcare OR "child care*" OR "day care*" OR daycare* OR "utility assistance" OR "medical legal") OR "health insurance" OR ("domestic violence" OR "family violence" OR (child NEAR/2 abuse) OR "elder abuse" OR "spous* abuse") OR (transport* OR commute OR commuting) OR ("interpersonal safety" OR (social NEAR/2 (interact* OR relationship OR isolat*)))) |
| 3 | TS=((((electronic OR medical OR personal) NEAR/2 ("medical record*" OR "health record*")) OR EHR OR EHRs OR EMR OR EMRs OR PHR OR PHRs)) |
| 4 | TS=(screen*) |
| 5 | #1 AND #2 AND #3 AND #4 |
|  | Limited to English language, 2015 - present |
|  | |
| **Database: Cochrane Central Register of Controlled Trials (CENTRAL) \| Wiley** | |
| 1 | ((((community OR neighborhood) NEAR/2 "health center*") OR CHC OR CHCs OR (health* NEAR/2 (clinic* OR service*))) OR ((primary NEAR/1 (healthcare OR "health care" OR care)) OR ((patient-centered OR "patient centered" OR patient-focused OR "patient focused") NEAR/1 care)) OR (hospital* OR ((inpatient* OR in-patient* OR outpatient* OR out-patient*) NEAR/3 (care OR service*))) OR ("ambulatory care" OR "urgent care" OR "clinic visit*" OR safety-net OR "safety net" OR "Federally Qualified Health Center" OR FQHC) OR ("emergency department*" OR "emergency room*" OR "emergency ward*")) |
| 2 | (("social determinant*" OR SDOH OR SDH) OR ((unmet OR under-met OR social OR economic) NEAR/1 need*) OR (education NEAR/2 (status OR achievement*)) OR (employ* OR unemploy* OR career* OR occupation* OR poverty OR income) OR (food NEAR/1 (suppl* OR assistance OR securit* OR insecurit*)) OR (housing OR homeless* OR rent*) OR ("public assistance" OR "social welfare" OR "social service*" OR medicare OR Medicaid OR childcare OR "child care*" OR "day care*" OR daycare* OR "utility assistance" OR "medical legal") OR "health insurance" OR ("domestic violence" OR "family violence" OR (child NEAR/2 abuse) OR "elder abuse" OR "spous* abuse") OR (transport* OR commute OR commuting) OR ("interpersonal safety" OR (social NEAR/2 (interact* OR relationship OR isolat*)))) |
| 3 | ((((electronic OR medical OR personal) NEAR/2 ("medical record*" OR "health record*")) OR EHR OR EHRs OR EMR OR EMRs OR PHR OR PHRs)) |
| 4 | (screen*) |
|  | #1 AND #2 AND #3 AND #4 |
|  | Limited to Clinical Trials, 2015 - present |
